# Nusinersen: the antisense oligonucleotide at the forefront of spinal muscular atrophy treatment

**DOI:** 10.1080/15476286.2026.2675858

**Published:** 2026-05-18

**Authors:** Natalia N. Singh, Eric W. Ottesen, Ravindra N. Singh

**Affiliations:** Department of Biomedical Sciences, Iowa State University, Ames, IA, USA

**Keywords:** Spinal muscular atrophy, SMA; survival motor neuron, SMN; ISS-N1, antisense oligonucleotide, 2‘-O-methoxyethyl modification, Spinraza^TM^, Nusinersen

## Abstract

Spinal muscular atrophy (SMA) is the first human disease to be treated with an antisense oligonucleotide (ASO) that restores the full coding sequence of a mRNA through splicing modulation. The therapeutic ASO nusinersen (marketed as Spinraza^TM^) targets intronic splicing silencer N1 (ISS-N1) located downstream of the predominantly skipped exon 7 of *Survival Motor Neuron 2* (*SMN2*) gene. The full-length transcript of *SMN2* codes for SMN, an essential housekeeping protein with a prominent role in RNA metabolism. The success of nusinersen could be attributed at least in part to the accessibility of ISS-N1 that was found to have a strong inhibitory effect on splicing of *SMN2* exon 7. Nusinersen has saved thousands of lives affected by SMA. However, limitations of an ASO-based therapy continue to emerge. Here we describe lessons learned from ASO-mediated splicing corrections in general and nusinersen in particular. Specific focus of this review is to discuss how information gleaned from the off-target effects of nusinersen could be utilized to develop next generation of ASO-based therapies with improved efficacies.

## Introduction

**Figure 1.**
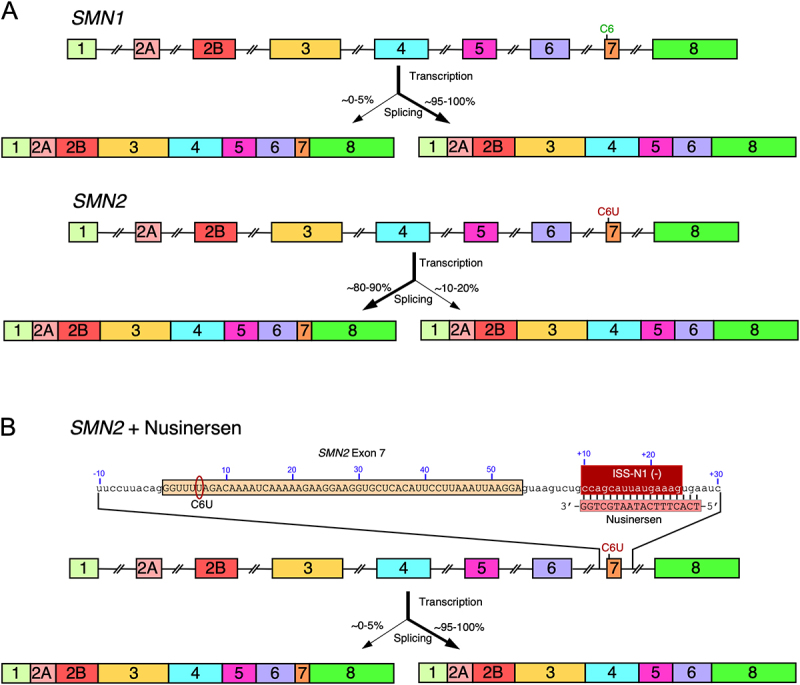
Alternative splicing of *SMN1* and *SMN2* transcripts and the effects of nusinersen. (A) Genomic overview of *SMN1* (upper) and *SMN2* (lower) genes. Exons are shown as colored boxes, introns as broken lines. C6 (*SMN1*) and C6U (*SMN2*) are labeled. After transcription, exon 7 of *SMN1* is fully included, generating full-length transcript, while exon 7 of *SMN2* is predominantly skipped, forming the *SMN2∆7* mRNA. (B) Effect of nusinsersen on splicing of *SMN2* exon 7. A closeup depicting the sequence of *SMN2* exon 7 and flanking intronic sequences is shown. Exon 7 is boxed and indicated with uppercase letters, while intronic sequences are shown with lowercase letters. Exonic positions are numbered relative to the 3′ss of exon 7. Upstream intronic positions are numbered with negative numbers relative to the 3′ss, while downstream intronic positions are numbered with positive numbers relative to the 5′ss. ISS-N1 is indicated in red. Nusinersen base pairs with ISS-N1 (base pairs indicated with black lines) and inhibits its negative effects, restoring predominant exon 7 inclusion.

Nusinersen (Spinraza^TM^) is an antisense oligonucleotide (ASO) approved in 2016 for the treatment of spinal muscular atrophy (SMA), the leading genetic cause of infant mortality [1–3]. SMA is a broad-spectrum disease and results from low levels of Survival Motor Neuron (SMN) protein due to deletions or mutations of *SMN1* gene [4–6]. *SMN2*, a nearly identical copy of *SMN1*, is universally present in humans due to duplication of a ~ 500 kb segment on chromosome 5 [7]. Here we use the term *SMN1/2* to refer to both *SMN1* and *SMN2*. *SMN1*/*2* possess similar promoter structure, harbour disproportionately high Alu content and contain nine exons, i.e. exons 1, 2A, 2B, 3, 4, 5, 6, 7 and 8 [8–11]. *SMN2* fails to compensate for the loss of *SMN1* due to skipping of exon 7 owing to a critical C-to-T mutation at the 6th position (C6U mutation in RNA) of exon 7 (Figure 1) [12,13]. *SMN2* mRNA lacking exon 7 produces SMNΔ7, a truncated protein with reduced stability and activity [14–18]. Considering *SMN2* is present in most SMA patients, prevention of *SMN2* exon 7 skipping has long been considered as one of the best options for SMA therapy [19,20]. The ultimate proof that the restoration of *SMN2* exon 7 inclusion could serve as a viable SMA therapy became available after the discovery of intronic splicing silencer N1 (ISS-N1) that led to the development of nusinersen (Figure 1) [1,3]. Importantly, the approval of nusinersen cemented the idea that an ASO could be employed to generate a functional protein by sequestration of intronic sequences.

The promise of developing new SMA therapies inspired investigators worldwide to interrogate potential mechanisms of *SMN1/2* exon 7 splicing regulation. During the past 25 years, more than 50 cis-elements, including structural elements and an equally large number of transacting factors participating in splicing modulation of the 54-nt long exon 7 have been discovered, expanding our understanding of *SMN1/2* exon 7 splicing regulation substantially [21–27]. *SMN1/2* exons 3 and 5 also undergo skipping, particularly under the conditions of oxidative stress, although limited attention has been paid towards uncovering the mechanisms of splicing regulation of these exons [28,29]. All internal exons of *SMN1/2* are divisible by three, hence skipping of any of them has no consequence on mRNA stability associated with nonsense-mediated decay (NMD). Exceptions to this rule include the incorporation of the cryptic exons due to exonization of intronic sequences and/or downstream exons generated due to failure of RNA polymerase II (pol II) to terminate transcription within exon 8 of *SMN1/2* [18,30]. Recent reports demonstrate co-transcriptional regulation of splicing of exons 3 and 7 of *SMN1/2* as skipping of exons 3 and 7 is augmented under the conditions of slow transcription elongation by pol II [31–33]. These findings support that the enhanced rate of *SMN2* transcription combined with the induction of *SMN2* exon 7 inclusion is a better therapeutic approach than a therapy based solely on promotion of *SMN2* exon 7 inclusion [31,34,35].

The success of ASO-based therapies depends on many factors, including the nature of the antisense target, potential off-target effects of the ASO on the transcriptome and proteome, as well as the pharmacokinetics and pharmacogenetics of the ASO. In order to confer stability and nuclease resistance in vivo, therapeutic ASOs are chemically modified at the sugar moiety, including 2′-*O*-methyl (OMe), 2′-*O*-methoxyethyl (MOE), and locked nucleic acids (LNA) modifications [36]. Most modified ASOs also incorporate phosphorothioate (PS) backbones to confer nuclease resistance as well as to achieve an improved pharmacodynamic profile [37]. Other modifications such as phosphorodiamidate morpholino oligonucleotides (PMOs) substitute the negatively charged backbone with a neutral one [36]. Nusinersen encompasses PS backbone and MOE modifications [3,38]. Several recent reports focus on real-world evidence on efficacy and safety of nusinersen treatment [39–42]. Here we review the journey leading to the discovery of ISS-N1 that served as the target for the development of nusinersen. We describe the unique context of ISS-N1 in regulation of *SMN1/2* exon 7 splicing. This review is inspired by a recent report uncovering enhancer-associated functions of ISS-N1-like sequences [43]. These ISS-N1-like sequences mediate unintended off-targets of nusinersen, primarily due to MOE modifications which appear to have unique tolerance for mismatches [43]. We discuss how meticulous design including oligonucleotide size and modifications could play a critical role in developing the next generation of ASO-based therapies. Finally, we compare nusinersen with other SMA therapies and conclude with future perspective.

## Discovery of ISS-N1 as a promising antisense target

**Figure 2.**
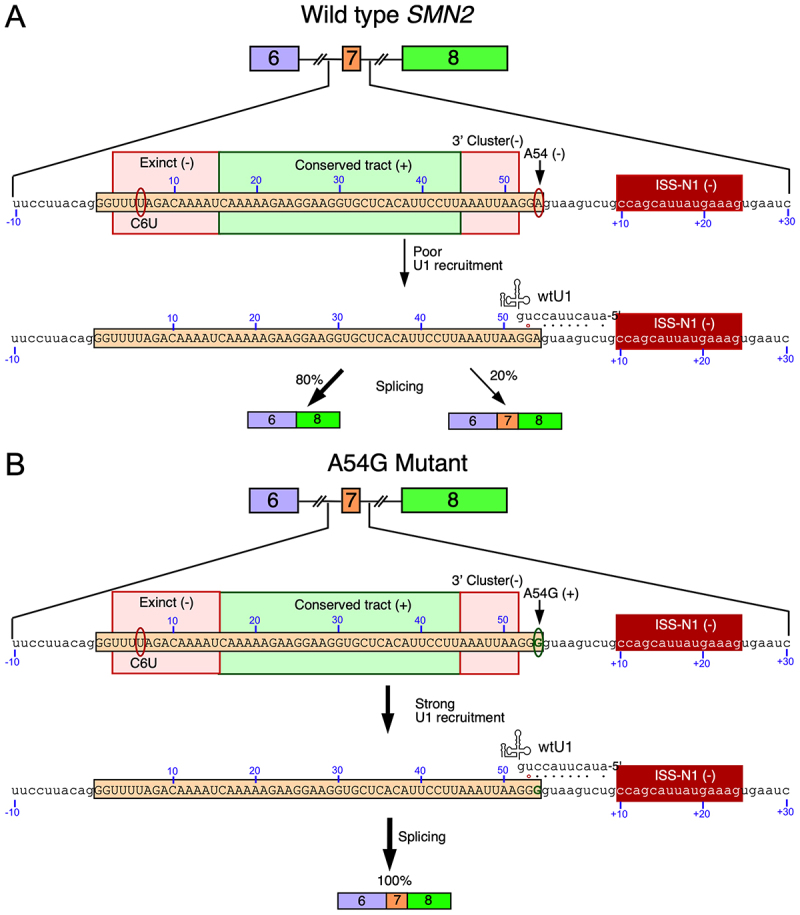
The impact of the last position of *SMN2* exon 7 on alternative splicing. (A) Splicing of wild type *SMN2* exon 7. Top panel portrays regulatory elements of *SMN2* exon 7. Negative sequences (exinct, 3′ cluster, and ISS-N1) identified by in vivo selection are boxed in red, while positive sequences (conserved tract) are boxed in green. C6U and A54 residues are circled in red. Other coloring and labeling are the same as in Figure 1B. Lower panel: base pairing of wild type U1 snRNA (wtU1) with the wild type *SMN2* exon 7 5′ss. Canonical base pairs are indicated with black dots, while G:U wobble base pairs are indicated with red circles. The a residue at the last position of exon 7 does not base pair with the cognate base in wtU1, potentially limiting recruitment. (B) Splicing of the A54G mutant of *SMN2* exon 7. Top panel portrays regulatory elements of *SMN2* exon 7 when A54G mutation is present. A54G is circled in green, other labels and colors are the same as (A). Lower panel: base pairing of wtU1 with the 5′ss of the A54G mutant of *SMN2*. mutation provides another strong G:C base pair between wtU1 and the 5′ss, fully restoring exon 7 inclusion.

Internal exons are defined by the 3′ splice site (3′ss) and the 5′ss at the beginning and the end of exons, respectively. Skipping of an exon is triggered by the inability of the splicing machinery to recognize both splice sites. In general, a weak 3′ss coupled with a strong 5′ss results in the retention of the upstream intron, whereas a weak 5′ss coupled with a strong 3′ss results in the retention of the downstream intron. *SMN1* minigene produces transcripts that retain intron 7 indicating that the 5′ss of exon 7 of *SMN1* is inherently weak [44]. Both *SMN1* and *SMN2* share identical 5′ss suggesting that the 5′ss of exon 7 of *SMN2* is also weak. Skipping of *SMN2* exon 7 points to the fact that *SMN2*-specific C6U mutation located close to the 3′ss weakens the 3′ss. Hence, early studies exclusively focused on the 3′ss, particularly on mechanisms by which C6U mutation triggers skipping of *SMN2* exon 7 [44–47]. It was even proposed that the enhanced inclusion of *SMN2* exon 7 through recruitment of splicing factors at the 3′ss employing bi-functional ASOs could potentially provide a therapeutic avenue [48,49]. An in vivo selection that examined the role of every exonic position in splicing of exon 7 revealed three regions involved in splicing regulation i.e. an extended inhibitory context (Exinct) towards the 5′-end of exon 7, a Conserved tract (positive region) in the middle of exon 7 which overlapped several previously reported positive regulatory elements [14,50–52] and an inhibitory 3′-Cluster towards the 3′-end of exon 7 (Figure 2) [50]. One of the most surprising findings of in vivo selection was the strong inhibitory impact of an adenosine residue (54A) at the last exonic position (Figure 2) [50]. A single A-to-G substitution at the last exonic position (A54G) fully restored *SMN2* exon 7 inclusion even in the absence of the positive regulatory elements, supporting that the strengthening of the 5′ss of exon 7 of *SMN2* would offer a promising therapeutic avenue [50]. Presence of A54G substitution strengthens the base pairing between the 5′ss of exon 7 and U1 snRNA, a component of U1 snRNP that defines the 5′ss. Hence, the strong stimulatory effect of A54G substitution supported that the suboptimal recruitment of U1 snRNP at the 5′ss of exon 7 is one of the major causes of skipping of *SMN2* exon 7 (Figure 2).

**Figure 3.**
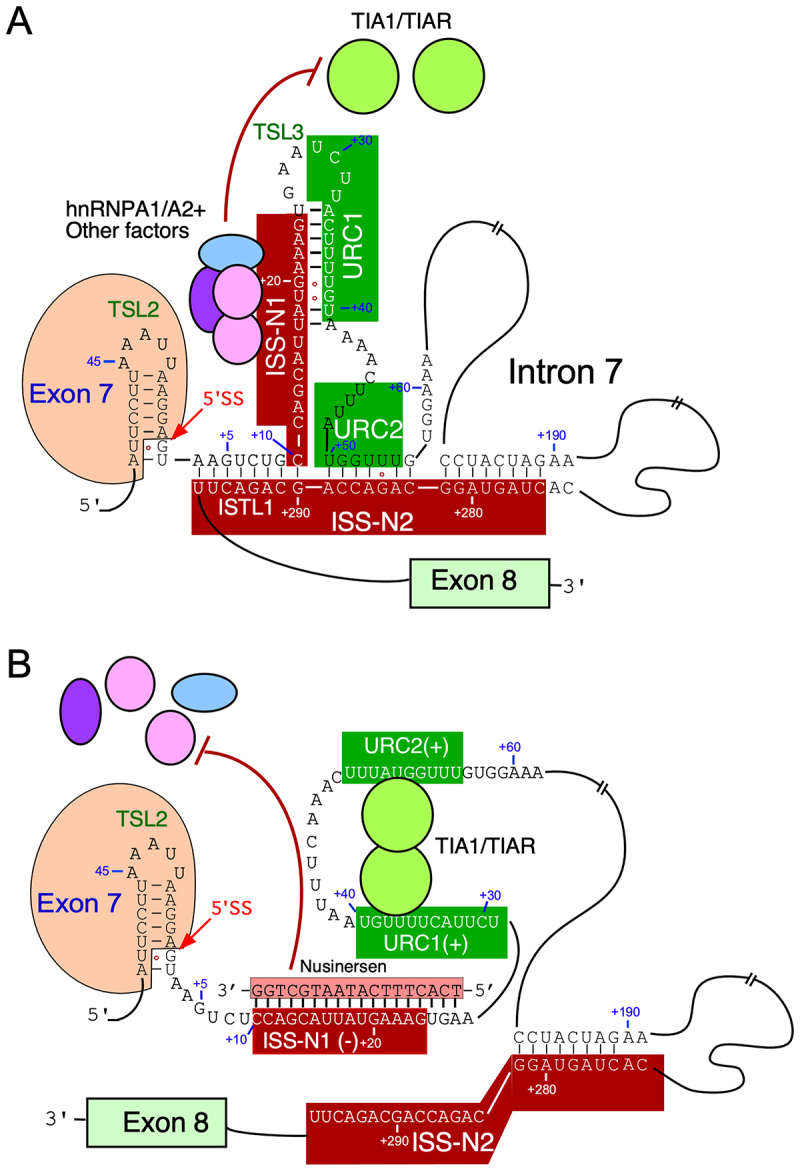
Proposed mechanism of nusinersen action is mediated by RNA structure and RNA-binding proteins. (A) Sequence and structural context of the 5′ss of *SMN2* exon 7. The last 17 bases of exon 7 are circled. Structural elements TSL2, TSL3, and ISTL1 are labeled. Canonical base pairs are indicated with black lines while G:U/G:T wobble base pairs are indicated with red circles. Negative sequence elements are boxed in red while positive elements URC1 and URC2 are boxed in green. Numbering is the same as in Figure 1B. hnRNPA1/A2 and potentially other protein factors interact with ISS-N1 and prevent interaction of positive splicing factors TIA1/TIAR. (B) Sequence and structural context of the *SMN2* exon 7 5′ss when nusinersen is present. Nusinersen base pairs with ISS-N1and prevents formation of both TSL3 and ISTL1 while also blocking the interaction of protein factors, allowing TIA1/TIAR to bind to URC1 and URC2.

The results of in vivo selection provided incentive for uncovering the mechanisms by which recruitment of U1 snRNP at the 5′ss of exon 7 could be enhanced. In a surprising revelation, substitutions and deletions within intron 7 region immediately downstream of the 5′ss of exon 7 produced a strong stimulatory effect on inclusion of *SMN2* exon 7 [53]. In particular, a 15-nt deletion from 10th to 24th positions of intron 7 fully restored *SMN2* exon 7 inclusion. This 15-nt sequence was termed as intronic splicing silencer N1 or ISS-N1 (Figure 3) [53]. Further, an ASO targeting ISS-N1 fully restored *SMN2* exon 7 inclusion, supporting the inhibitory nature of ISS-N1 [53]. Confirming the target specificity of the ASO, mutations within the target sequence or the ASO abrogated its stimulatory effect. Follow-up experiments in type 1 SMA patient fibroblasts that contain only *SMN2* validated the stimulatory effect of the ISS-N1-targeting ASO on inclusion of *SMN2* exon 7 and levels of SMN [53]. In a rare finding, the strong stimulatory effect of the ISS-N1-targeting ASO was captured even at the low concentration of 5 nM, making ISS-N1 as one of the most desirable targets for the therapeutic development [53].

## Mechanism of ISS-N1-targeting ASO in splicing modulation

ISS-N1 is positioned immediately downstream of the 5′ss of exon 7 and it has been proposed that the interaction of ISS-N1 with hnRNP A1/2 prevents recruitment of U1 snRNP at the 5′ss exon 7 (Figure 3) [54,55]. Supporting this argument, depletion of hnRNP A1/A2 stimulates inclusion of *SMN2* exon 7 [56]. However, findings of depletion experiments should be treated with caution as they do not serve as a definitive proof of direct interactions of hnRNP A1/A2 with ISS-N1, since additional hnRNP A1/2 binding sites within exon 7 and intron 7 of *SMN2* have been reported [47,57,58]. It is possible that multiple interactions of hnRNP A1/A2 loop-out *SMN2* exon 7 as proposed for other skipped exons [59,60]. Depletion of several splicing factors, including SRSF2, SRSF3, SRSF4, SRSF5, SRSF6, SRSF7, SRSF11 and hnRNP U, are known to stimulate inclusion of *SMN2* exon 7 [61]. It is possible that one or more of these factors exert their inhibitory effect on *SMN2* exon 7 splicing through interaction with ISS-N1. Uridine-rich clusters/sequences referred to as URC1 and URC2 immediately downstream of ISS-N1 interact with TIA1/TIAR that are known to promote recruitment of U1 snRNP at the 5′ss of an exon (Figure 3) [62,63]. Consistently, overexpression of TIA1/TIAR fully restore *SMN2* exon 7 inclusion in the context of minigene [62]. Also, deletion of sequences corresponding to TIA1/TIAR binding site increase skipping of *SMN2* exon 7 [62]. Therefore, it is possible that the interaction of inhibitory factors with ISS-N1 sequesters binding sites of TIA1/TIAR and consequently adversely affects the recruitment of U1 snRNP to the 5′ss of exon 7.

The 5′ss of *SMN2* exon 7 is partially sequestered by terminal stem-loop 2 (TSL2), an inhibitory RNA structure that hinders the recruitment of U1 snRNP (Figure 3) [64]. Consistently, disruption of TSL2 or strengthening of base pairing between the 5′ss of exon 7 and U1 snRNA has been shown to promote *SMN2* exon 7 inclusion [64]. Structure probing of the entire *SMN2* intron 7 revealed several internal stems formed through long-distance interactions or ISTLs [56]. One of these structures, ISTL1, sequesters a portion of the 5′ss. In other words, the combined structures of TSL2 and ISTL1 fully sequester the 5′ss of exon 7 making it completely inaccessible for U1 snRNP (Figure 3) [65]. While the 5′-strand of ISTL1 is provided by the 5′ss located at the beginning of intron 7, the 3′-strand of ISTL1 is located in the middle of intron 7, making ISTL1 one of the rare splicing regulatory structures confirmed to be formed by a long-distance interaction. Confirming the inhibitory nature of ISTL1, ASOs disrupting ISTL1 by annealing to either strand promote *SMN2* exon 7 inclusion [56,66]. These ASOs also show therapeutic efficacy in mouse models of SMA [67,68]. The C residue at the 10th intronic position (^10^C) occupies the first position of ISS-N1 and happens to be last residue of the 5′-strand of ISTL1. A 14mer ASO that sequestered the last 14 residues of ISS-N1 but not the ^10^C residue enhanced skipping of *SMN2* exon 7, whereas an 8-mer ASO that sequestered just first five residues of ISS-N1 in addition to three upstream residues stimulated *SMN2* exon 7 inclusion [56,66,69]. These findings underscore that the disruption of the inhibitory RNA structure associated with ISS-N1 is the driving contributor of the stimulatory effect of an ISS-N1-targeting ASO.

## Engineered U1 snRNP targeting ISS-N1 promote *SMN2* exon 7 inclusion

**Figure 4.**
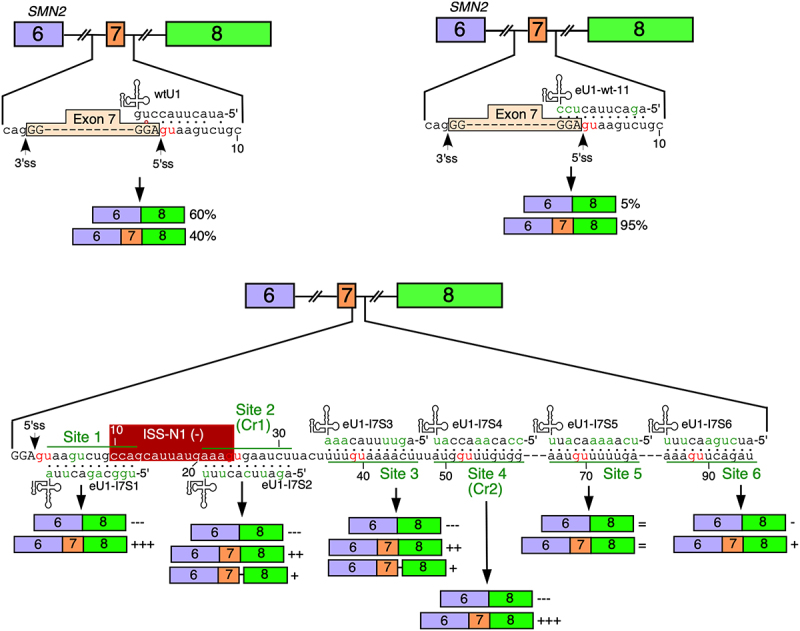
Engineered U1 (eU1) snRNAs targeting sequences at and near the 5′ss restore *SMN2* exon 7 inclusion. Top left: interaction of wild type U1 snRNA (wtU1) with the *SMN2* exon 7 5′ss. Canonical base pairs are indicated with black dots while G:U wobble base pairs are indicated with red circles. Top right: eU1 restoring full complementarity to the 5′ss of *SMN2* exon 7 predominantly restores exon 7 inclusion. Lower panel: six eU1s targeting different GU dinucleotides have different impacts of *SMN2* exon 7 splicing. ISS-N1 is indicated with a red box. Isoforms are shown below the base pairing diagram, ‘-’ indicates decreased isoform amount, ‘+’ indicates increased isoform amount. Two eU1s trigger usage of a cryptic 5′ss in intron 7 at position 23 (Cr1), which is indicated in the diagram with a short line in between exon 7 and exon 8 boxes.

The most well studied role of U1 snRNP in modulation of splicing is the selection of the 5′ss through direct interaction of U1 snRNA with the last three and the first six residues of an exon and intron, respectively [70]. Perfect complementarity between the 5′ss and U1 snRNA is not an absolute requirement for the U1 snRNP-mediated definition of the 5′ss, as gaps and bulges within the RNA:RNA duplex formed between the 5′ss and U1 snRNA are tolerated [71]. There is also evidence to support that the recruitment of U1 snRNP at cryptic splice sites suppresses inclusion of cryptic exons and facilitates accurate removal of introns [72]. A splicing-independent role of U1 snRNP is the modulation of transcript length through interactions with sequences away from the 5′ss [73]. U1 snRNA also forms another U1 snRNP like complex called U1-TAF15 snRNP that interacts with chromatin [74]. Recent reports demonstrate that engineered U1 snRNAs (eU1s) with increased complementarity with the 5′ss-like sequences away from the natural 5′ss, including at ISS-N1 promote usage of the natural 5′ss, including the 5′ss of all *SMN1/2* exons (Figure 4) [33,75–77]. The finding that eU1s targeting ISS-N1 or downstream sequences restore *SMN2* exon 7 inclusion supports that the inhibitory contexts enabled by TSL2, ISTL1 and ISS-N1 could be fully abrogated by alternative mechanisms. Future studies will determine if both U1 snRNP and U1-TAF15 snRNP play equal role in selection of the 5′ss from a distance.

## ISS-N1 as the most studied antisense target for splicing modulation

**Figure 5.**
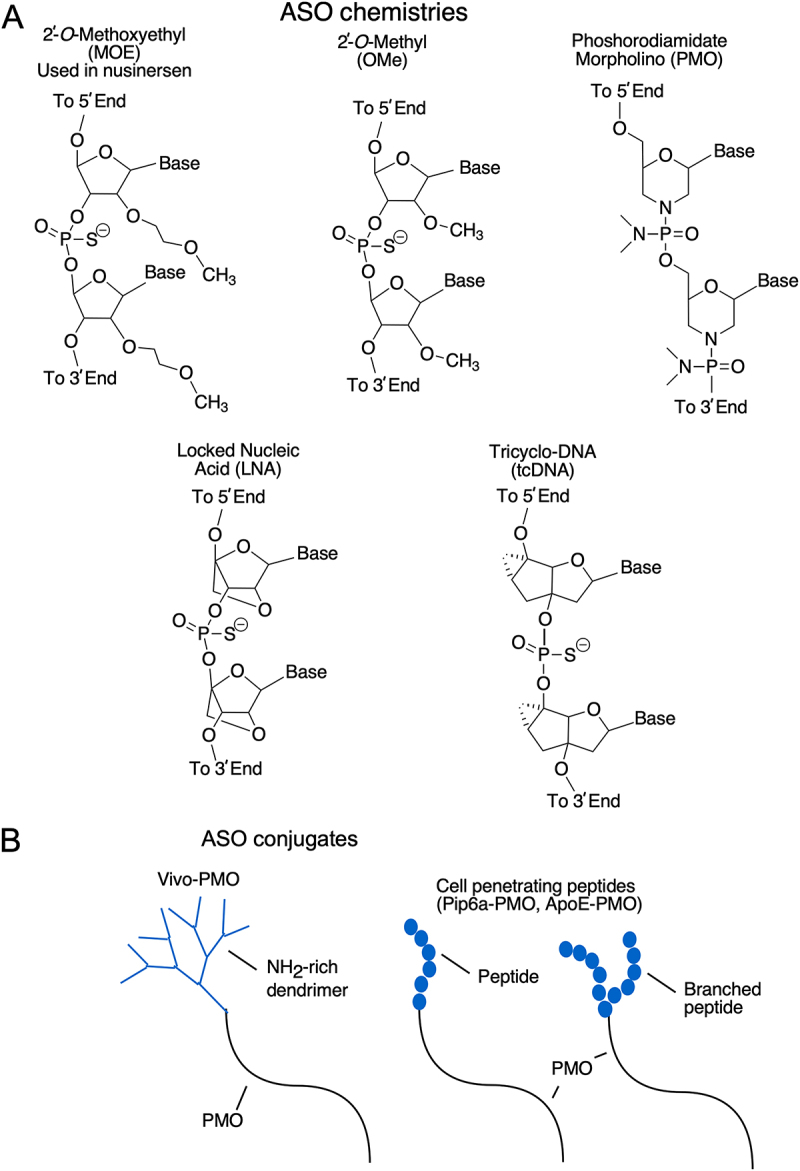
ISS-N1 has been the target of a wide range of ASO chemistries and ASOs carrying terminal modifications. (A) The chemical structures of five different ASO chemistries that have all been used for *SMN2* exon 7 splicing correction. (B) Graphical overview of PMO ASOs with different end modifications to facilitate transport into cells and across the BBB.

The first study on ISS-N1 published in 2006 by Singh lab at University of Massachusetts Medical School, MA, USA, employed 20mer ASOs encompassing PS backbone and OMe modifications (Figure 5) [53]. A subsequent study published in 2008 by Krainer lab at Cold Spring Harbor Laboratory, NY, USA in collaboration with Ionis Pharmaceuticals (previously ISIS Pharmaceuticals) employing 18mer ASOs encompassing PS backbone and MOE modifications confirmed that ISS-N1-targeting ASO, dubbed as ASO 10–27, was most efficacious in promotion of *SMN2* exon 7 inclusion as compared to other ASOs targeting different sequences of *SMN2* (Figure 5) [54]. An in vivo study employing ASO 10–27 showed unprecedented efficacy in extending the life expectancy of SMA mice [78]. These findings led to clinical trials of ASO 10–27 (renamed as nusinersen) that was subsequently approved by FDA as the first SMA drug in 2016 [38]. Several groups of investigators, including Burghes lab at The Ohio State University, OH, USA, Muntoni lab at the University College of London, UK, Fletcher and Wilton labs at the University of Western Australia, employed 20mer and longer ISS-N1-targeting PMOs and demonstrated their very high in vivo efficacy (Figure 5) [79–81]. Additional ISS-N1-targeting ASOs encompassing other modifications such as locked nucleic acids (LNAs), and tri-cyclo DNA (tcDNA) have shown high efficacies in restoration of *SMN2* exon 7 inclusion in cell-based systems and/or in mouse models of SMN as well (Figure 5) [69,82–84]. Pilot studies using various cell-penetrating peptides and NH_2_-rich dendrimer (vivo morpholino) conjugated to PMOs to encourage transport across the blood-brain barrier have also shown promise [85,86]. Based on the studies published thus far, ISS-N1 remains the most studied antisense target for splicing modulation.

## Biodistribution of ISS-N1-targeting ASOs in mouse models of SMA

SMN is a housekeeping protein with multiple cellular functions including cytoskeletal maintenance, DNA replication, DNA repair, RNA metabolism, signal transduction and macromolecular trafficking [87]. Low SMN level affects all tissues, including brain, heart, gut, kidney, liver, lung, muscle, pancreas, spinal cord and testis [88–100]. Hence, it is important that therapeutic intervention in SMA ensures body-wide restoration of SMN. A study conducted in a severe mouse model of SMA showed the best efficacy of ASO 10–27 (nusinersen) when two doses of ASO were administered subcutaneously (SC), one at postnatal day zero (P0) and the other at P3 [78]. The body-wide distribution of ASO 10–27 was confirmed by monitoring the splicing changes. Indeed, subcutaneous administration of ASO 10–27 at P0 and P3, before full establishment of the blood-brain barrier (BBB), showed substantial restoration of *SMN2* exon 7 in spinal cord, heart, brain, muscle, liver and kidney at the highest ASO concentration used (160 μg/g body weight) [78]. The median survival of the severe SMA mice at the highest concentration of the SC-administered ASO 10–27 increased from 10 days to 248 days [78]. This rate of survival of the severe SMA mice upon SC-administration of ASO 10–27 was better than the SMN restoration using adeno-associated virus in the less severe SMA mouse model [101,102]. Two of the 14 mice that received high SC dose of ASO 10–27 survived beyond 500 days [78]. Intracerebroventricular (ICV) delivery of ASO 10–27 had a modest effect on survival due to the lack of restoration of SMN in peripheral tissues, although investigators used low ASO doses likely due to brain-associated toxicity expected from the charged phosphorothioate backbone [78,103]. Supporting this argument, better efficacies of ICV administrations were observed with ISS-N1-targeting PMOs that encompass neutral backbone [79–81].

Despite the finding that ASO 10–27 superbly performed upon SC administration in severe SMA mice, developers of nusinersen decided to employ intrathecal administration in SMA patients. This decision was made in part because the findings using SMA mouse model are not likely to hold true in case of SMA patients because human brain is impermeable to oligonucleotides. A side-by-side comparison using severe neonatal SMA mice SC-administered with the similar doses of ISS-N1-targeting ASOs of identical sizes showed better efficacy for MOE/PS chemistry than PMO one [104]. However, given the fact that SMA patients receive nusinersen through intrathecal administration, a side-by-side comparison of the efficacy of ASOs employing ICV administration would have been more informative. Severe SMA mice have been shown to have prolonged lifespan upon receiving an ISS-N1-targeting PMO through intravenous (IV) administration [81]. IV administration of an ASO provides a high probability of body-wide distribution with the exception of the delivery across the BBB. Several strategies to enable transportation of ISS-N1-targeting PMOs across BBB upon IV administration have been reported [85,86,105,106]. These findings lay strong foundation for developing the next generation of improved ASO-based therapies for SMA.

## Clinical trials of nusinersen and therapeutic regimen

The phase 1 clinical trial of nusinersen (ISIS-SMNRx) was launched by IONIS Pharmaceuticals (formerly ISIS Pharmaceuticals) in 2011, where ASO was administered intrathecally to SMA types 2 and 3 patients aged 2–14 years in an open-label study (NCT01494701; NCT01780246). Nusinersen was well tolerated, and the intrathecal administration was considered safe for SMA children [107]. Subsequent phase 2 open-label clinical trial evaluated safety, tolerability, pharmacokinetics, and clinical efficacy of multiple intrathecal doses of nusinersen (6 mg and 12 mg dose equivalents) in SMA infants aged 3 weeks −7 months (NCT01839656). Nusinersen showed acceptable safety, tolerability, and pharmacological properties [108]. The phase 3 randomized, double-blinded, and sham-controlled clinical trial examined the efficacy and safety of nusinersen in infants with SMA (NCT02193074). SMA infants receiving nusinersen had a higher rate of survival than those receiving the sham control [109]. Based on the findings of phase 3 clinical trial, Food and Drug Administration (FDA) of USA approved nusinersen in 2016 [1–3]. Subsequently, nusinersen was approved in other countries including Europe, Japan, Brazil, Argentina, Russia, Turkey, Mexico, and China. Additional clinical trials of nusinersen have shown mixed results and has been recently reviewed [41].

Current practice of ASO therapy of SMA involves intrathecal administration of multiple 12 mg/5 ml doses of nusinersen to all types of patients irrespective of age. Initial three administrations are performed at 14-day intervals followed by a 4th administration after 30 days. Subsequent administrations are performed at 4-month intervals. As of March 2026, more than 9000 children and 5,300 adults have been treated using the above regimen. In March 2026, FDA approved high dose intrathecal administrations of nusinersen. As per new regimen, new patients will first receive two 50 mg/5 ml doses separated by 14 days, followed by maintenance doses of 28 mg/5 ml every four months. Patients currently on low-dose regimen will have an option to switch to the high dose regimen. In this case, they will initially receive a single 50 mg/5 ml dose followed by maintenance doses of 28 mg/5 ml every four months. The better efficacy of the high-dose regimen could be due to sustained availability of drug and/or improved distribution across tissues. Adverse effects of nusinersen include pyrexia, vomiting, constipation and elevated markers of kidney dysfunction [110–112]. To mitigate some of these concerns, an improved version of nusinersen is in clinical trial by Biogen, which currently markets nusinersen [41].

### A subset of patients treated with nusinersen express high levels of a circular RNA of SMN1/2

**Figure 6.**
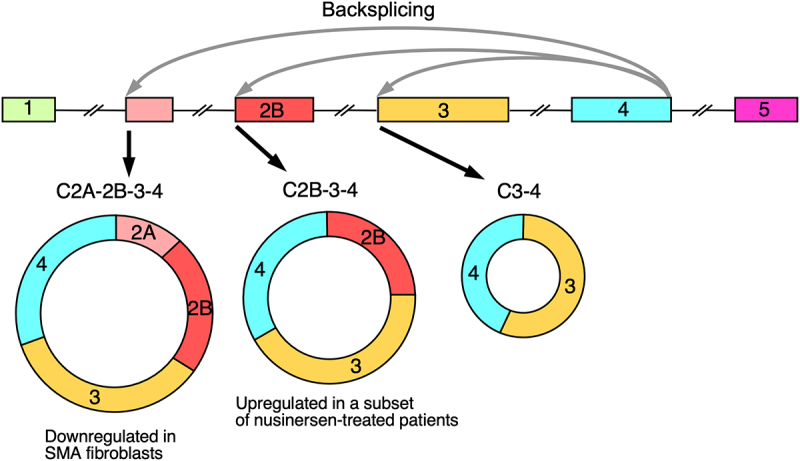
Mechanism of generation of circRnas from *SMN1/2*. *SMN1/2* genes are shown. Gray arrows represent backsplicing events generating circRNAs. The three most prevalent *SMN1/2* circRNAs are shown: C2A-2B-3–4, C2B-3–4, and C3-4. All three circRnas are cross-regulated considering they use the same 5′ss of exon 4.

Circular RNAs (circRNAs) are produced in cells of all living organisms and are more stable than linear RNAs [113]. Usually, circRNA biogenesis involves backsplicing, in which a downstream 5′ss pairs with an upstream 3′ss [114]. This splice-site pairing is facilitated by RNA-binding proteins as well as by RNA structures [115]. In humans, these RNA structures are often formed between inverted Alu repeats [116]. Functions of circRNAs include sponging of microRNAs (miRNAs), sequestration of proteins, transcription regulation and novel protein production [117–120]. CircRNAs are aberrantly expressed in many disease conditions; hence, they offer novel avenues for diagnosis and therapy [121–123]. Consistent with the unusually high content of Alu elements (~40%) within *SMN1/2* [10,124,125], a vast repertoire of circRNAs is generated from *SMN1/2* [30,126,127]. C2A-2B-3–4, C2B-3–4 and C3-4 are among the most abundant ones, produced by backsplicing of the 5′ss of exon 4 paired with the 3′ss of the upstream exons 2A, 2B and 3, respectively (Figure 6) [30,127]. Interestingly, C2B-3–4 and C3-4 are not expressed in mouse, supporting that these circRNAs are specific to primates [30].

Although functions of most circRNAs of *SMN1/2* remain unknown, C2A-2B-3–4 has been shown to affect expression of ~15% genes including those associated with chromatin remodelling, transcription, spliceosome function, ribosome biogenesis, lipid metabolism, cytoskeletal formation, cell proliferation and neuromuscular junction formation [128]. C2A-2B-3–4 is downregulated in type 1 SMA patient cells and its expression is cross-regulated by C2B-3–4 and C3-4 [30]. A recent report identified extremely high levels of C2B-3–4, also referred as circ4-2b-3 (up to 1000-fold overexpression), in a subset of type I SMA patients treated with nusinersen [129]. Most importantly, overexpression of C2B-3–4 well correlated with the improved motor outcomes in SMA patients [129]. However, it is not known if high levels of C2B-3–4 improve the efficacy of nusinersen or vice versa. Interestingly, high concentration of an ISS-N1-targeting ASO has been shown to promote skipping of *SMN1/2* exon 3 and inclusion of exon 6B, an Alu-derived cryptic exon [66,130]. It is not known if the enhanced skipping of exon 3 of *SMN1/2* is associated with generation of C2B-3–4.

## Off-target effects of ISS-N1-targeting ASOs

**Figure 7.**
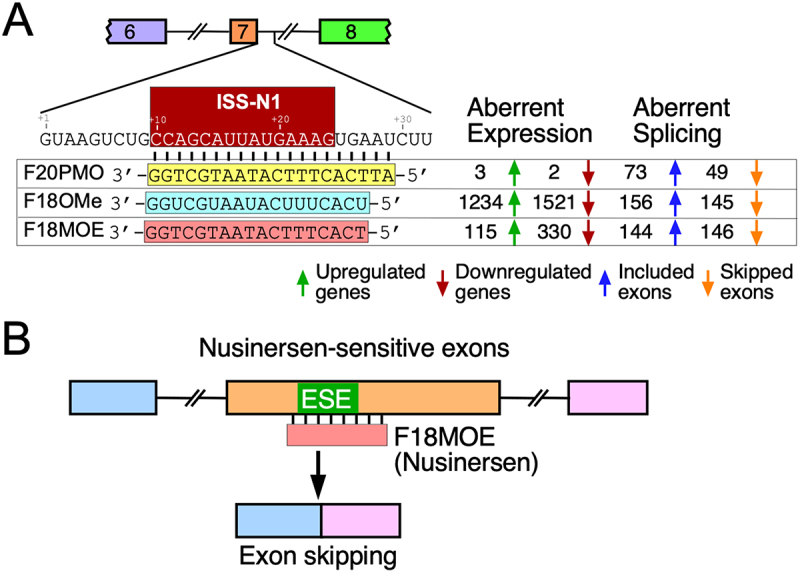
Overview of transcriptome-wide off-target effects of three ISS-N1-targeting ASOs. Base pairing of ASOs to ISS-N1 are shown, with the magnitude of off-target effects indicated to the right. Upregulated genes are indicated with green up arrows, while downregulated genes are indicated with red down arrows. Increased exon inclusion events are indicated with blue up arrows, while increased exon skipping is indicated with orange down arrows. (B) Mechanism of off-target effects of F18MOE/nusinersen on exon skipping. ASO binds to exonic sequences of off-target exons, masking ISS-N1-like exonic splicing enhancers (ESEs) and triggering exon skipping.

ASOs produce sequence-independent and sequence-dependent off-target effects. While a sequence-independent off-target effect is exerted through interactions of ASO with cellular proteins, sequence-dependent off-target effect is realized through direct base pairing of the ASO to a sequence other than the intended target. The PS backbone, which is present in nusinersen, is known to have sequence-independent effects as it interacts with cellular proteins, often sequestering them in nuclear aggregates [37,131,132]. The severity of sequence-independent effects can vary greatly depending on the ASO sequence [131]. Independent reports confirm that the ISS-N1-targeting ASOs encompassing PS/OMe modifications cause massive perturbation of the transcriptome [130,131]. A recent study compared the off-target effects of three ISS-N1-targeting ASOs with different chemistries such as F18MOE, F18OMe and F20PMO [43]. While F18MOE had the identical sequence and modifications to that of the nusinersen, F18OMe presented an 18mer ASO encompassing PS/OMe modifications and F20PMO represented a 20mer PMO. F18OMe affected the expression of 2755 genes (out of 27,369 expressed genes that were analysed) and F18MOE affected the expression of 445 genes (Figure 7) [43]. Interestingly, only ~10% of genes (272 out of 2755) were affected by F18OMe in a sequence-dependent manner. Among 272 genes affected by F18OMe in a sequence-dependent manner, 125 were upregulated and 147 were downregulated. In contrast, ~42% of genes (185 out of 445) were affected by F18MOE in a sequence-dependent manner. Among 185 genes affected by F18MOE in a sequence-dependent manner, 42 were upregulated and 143 were downregulated. F20PMO had the fewest off-target effects as only 5 genes were impacted [43]. Among genes that were found to be significantly downregulated at all concentrations of nusinersen in a sequence-dependent manner were *WDR70*, *CAPN7*, and *MGME1* [43]. Several genes, including *PPAT*, *TMEM97*, *MMP16* and *MICAL2* were upregulated at all concentrations of nusinersen in a sequence-independent manner [43]. These findings underscored the adverse effect of high concentration of nusinersen on many cellular processes including DNA replication and repair, RNA metabolism and protein turnover.

The recent study also analysed aberrant splicing triggered by nusinersen and captured off-target effects on 146 skipping and 144 inclusion events (Figure 7) [43]. Prominent among nusinersen-specific aberrant splicing events were skipping of *POLR2H* exon 2, *PITHD1* exon 2, *SERPINB7* exon 4, *RTTN* exon 4, *REV3L* exon 9, *PRKRA* exon 2, *GOLGA4* exon 4, and *PAK1* exon 2. Interestingly, ISS-N1 targeting ASOs encompassing other modifications, namely F18OMe and F20PMO, had no effect of splicing of the above exons. Subsequent experiments revealed the presence of a broad spectrum of nusinersen-responsive elements in the exonic sequences. The experiments demonstrated high tolerance for mismatch base pairing between nusinersen and ISS-N1-like sequences. Nusinersen-responsive elements were found to be portable in different contexts and serve as enhancers and silencers when present within exons and introns, respectively. The findings revealed the unexpected role of ISS-N1-like sequences as enhancers when present in the context of an exon (Figure 7) [43]. Although interaction of hnRNP A1/A2 with ISS-N1 has been proposed as a potential mechanism of the negative effect of ISS-N1, other factors appeared to be involved in interaction with the nusinersen-responsive elements.

## Truncated nusinersen produces reduced off-target effects

**Figure 8.**
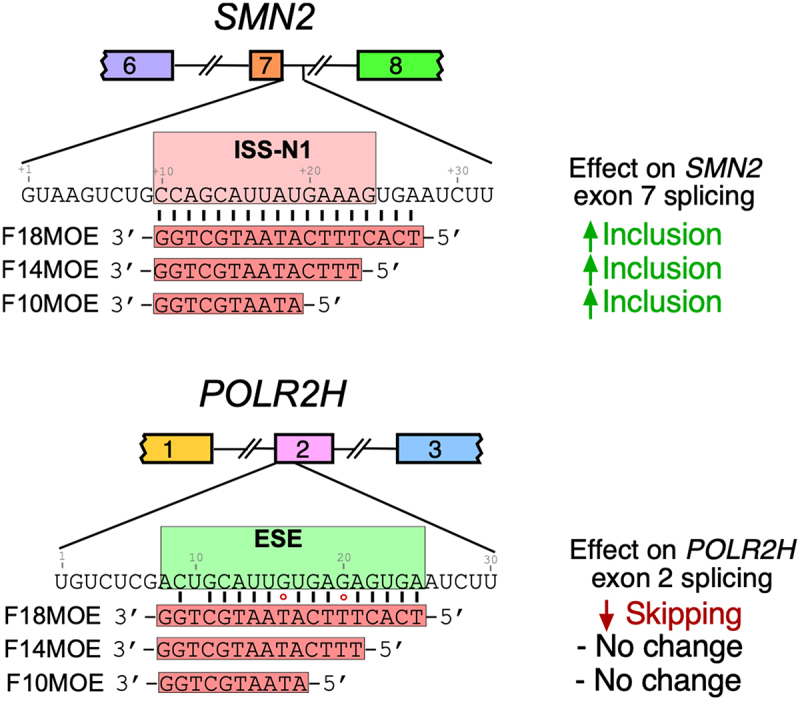
Shorter ASOs minimize hybridization-mediated off-target effects of nusinersen. Left: base pairing of F18MOE/nusinersen as well as shorter 14-mer and 10-mer sequences to ISS-N1 downstream of *SMN2* exon 7. All three ASOs trigger increased inclusion of *SMN2* exon 7. Right: base pairing of F18MOE/nusinersen as well as shorter 14-mer and 10-mer sequences to the off-target *POLR2H* exon 2. Due to mismatches and wobble base pairs between ASOs and the off-target exon, only the full-length 18mer has any effect on splicing.

Prior studies show that shorter splice-correcting ASOs tend to have fewer hybridization-dependent off-target effects [66,67,133]. This is likely due to low tolerance for mismatch base pairing by shorter ASOs. This hypothesis was tested and confirmed in the context of *POLR2H* exon 2 that harbours a nusinersen-responsive element. For instance, truncation of 18mer nusinersen from either end to a 14mer ASO fully eliminated the off-target effect on *POLR2H* exon 2 splicing (Figure 8) [43]. Notably, the 14mer ASOs regained the inhibitory effect on *POLR2H* exon 2 splicing when full complementarity with the nusinersen-responsive element was restored. Further truncations of nusinersen to 10mer ASOs produced mixed results. For example, while the 10mer ASO with full complementarity towards the 5′-end of the nusinersen-responsive element triggered skipping of *POLR2H* exon 2, another 10mer ASO with full complementarity with the 3′-end of the nusinersen-responsive element had no effect [43]. These results supported the presence of the critical enhancer motifs towards the 5′-end of the nusinersen-responsive element, although it is also possible that the annealing properties of the different portions of the nusinersen-responsive element play a role as well. Truncation of nusinersen also suppressed the off-target effect on expression of several transcripts that were downregulated by nusinersen [43]. Findings confirmed that the large size of nusinersen contributes towards the broad perturbation of the transcriptome.

## ISS-N1-targeting ASOs with mixed modification reduce off-target effects

**Figure 9.**
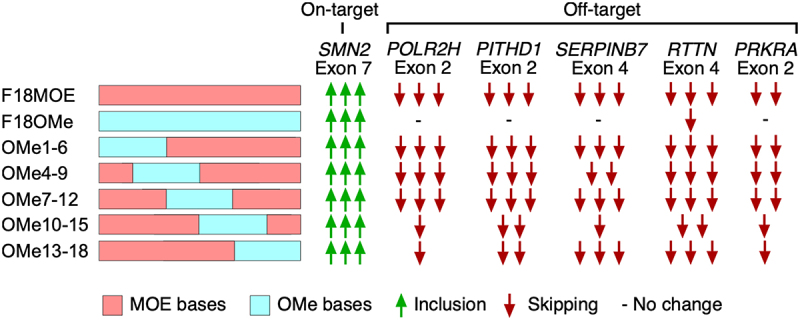
ASOs of mixed chemistry encompassing MOE and OMe residues partially reduce off-target effects on exon skipping. Different ASOs used for the study are shown at the left: pink regions indicate MOE while blue regions indicate OMe bases. ASOs are oriented from 3′ to 5′ left to right. Green up arrows indicate exon inclusion, while red down arrows portray exon skipping, with different numbers of arrows indicating strength of effect. All ASOs equally improve *SMN2* exon 7 inclusion. Across five different off-target exons, effects of mixed chemistry ASOs varied.

The study tested MOE-to-OMe substitutions at different positions of nusinersen and monitored the off-target effect on splicing of *POLR2H* exon 2. While all ASOs with mixed modifications retained the stimulatory effect on inclusion of *SMN2* exon 7, many of them reduced or eliminated off-target effect on splicing of *POLR2H* exon 2 (Figure 9) [43]. The finding supported that MOE modifications towards the 5′-half of nusinersen is critical for triggering *POLR2H* exon 2 skipping. Maximum reduction of the off-target effect was observed when MOE-to-OMe substitutions were inserted from 4th to 6th positions from the 5′-end of nusinersen [43]. It is likely that the high tolerance of MOE modification for a wobble base pairing at the 6th position from the 5′-end of nusinersen contributes towards skipping of *POLR2H* exon 2. However, MOE-to-OMe substitutions at other positions of nusinersen also reduced the off-target effect on *POLR2H* exon 2 splicing [43]. The findings supported that the off-target effect of nusinersen on *POLR2H* exon 2 splicing is mediated through multiple stretches of MOE modifications. Findings also confirmed that MOE modifications in different regions of nusinersen are associated with the off-target effects on expression and/or splicing of other genes.

## Comparison of nusinersen with other approved therapies of SMA

Subsequent to the approval of nusinersen in 2016, onasemnogene abeparvovec (Zolgensma) and risdiplam were approved for the treatment of SMA in 2019 and 2020, respectively [134,135]. Zolgensma is a gene therapy-based approach that uses AAV9 vector delivered intravenously [136]. It has an advantage of a single administration therapy, and many SMA patients are currently benefiting from Zolgensma. Adverse effects of onasemnogene abeparvovec include hepatotoxicity, thrombocytopenia, thrombotic microangiopathy, respiratory and dorsal root ganglion (DRG) toxicity [137]. To mitigate these concerns, intrathecal delivery of onasemnogene abeparvovec (itvisma) has been recently approved. Unlike splicing modulating SMA therapies that depend upon expression of endogenous *SMN2*, gene therapy utilizes hybrid cytomegalovirus (CMV) enhancer/chicken β-actin (CB or CBA) promoter to drive overexpression of the *SMN1*. Overexpression of SMN using AAV9 vector has been found to produce toxic effects in brain in a mouse study [138]. Therefore, it would be important to monitor the long-term consequences of intrathecal administration of itvisma. Risdiplam is a small molecule, and, similar to nusinersen, prevents *SMN2* exon 7 skipping [139]. Risdiplam offers the advantage of oral administration and has provided desired therapeutic benefits to SMA patients [135]. Compared to nusinersen, high concentrations of risdiplam trigger greater degree of transcriptome-wide perturbations [140]. Adverse effects of risdiplam include fever, diarrhoea, nausea, constipation, skin rash, ulcers in the mouth and oral area, urinary tract infection and joint pain [141,142]. Thus far, there has not been any clinical trial comparing head-to-head the effectiveness and adverse effects of the approved therapies of SMA.

In a recent observational study conducted on the matched cohorts from the French National SMA Registry, gene therapy was associated with lower incidence of unsatisfactory clinical response than nusinersen [143]. Findings suggested gene therapy as a promising first-line option for type 1 SMA patients with a high risk of bulbar and respiratory impairments. Yet most ventilatory supports were needed during the first year of treatment with nusinersen or gene therapy. In a different study, SMA patients showed improved motor gains when switched to gene therapy from nusinersen or risdiplam treatments [144]. The most pressing rationale for such switching was the reduced treatment burden due to one-time administration in case of gene therapy. Notably, switching to gene therapy increased the risk of liver enzyme elevation, systemic immune reactions and organ toxicities [144]. There have been incidences when patients switched from nusinersen to risdiplam to avoid pain and anxiety associated with lumber punctures [145,146]. Particularly, study found favourable effects of switching to risdiplam in older patients [145]. Progress thus far indicate that the availability of multiple therapeutic options would benefit different cohort of SMA patients at different stages of the disease progression. However, consensus is also emerging that the available therapies of SMA do not fully meet patients’ needs as they have limitations due to low efficacy and/or adverse effects [112,147–153]. Additional concerns relate to the fact that none of the available therapies of SMA are cost-effective compared to the best support therapy [154]. Study conducted on SMA type I patients in the Netherlands found gene therapy more cost effective than nusinersen after 8.25 years of treatment [155]. Based on a limited study in Europe, cost of risdiplam treatment was assessed to be significantly lower than nusinersen [156]. While percentage of nonadherence linked to high cost of nusinersen remains unknown, a study points to the low adherence and persistence to nusinersen treatment [157]. On the other hand, cost-related nonadherence has been reported for patients receiving risdiplam [158].

## Concluding remarks

Since its approval about 10 years ago, nusinersen has had a profound impact on SMA therapy as more than 14,000 patients worldwide have been treated with this drug. Many protocols developed to evaluate the therapeutic efficacy of nusinersen remain as the basis for the approval of subsequent drugs of SMA. Approval of nusinersen also served as a strong impetus for the development of ASO-based therapies of other diseases [159,160]. Since the discovery of ISS-N1 more than two decades ago, no comparable target has emerged for ASO-mediated splicing correction in SMA. While all available SMA therapies have their own advantages and limitations, tremendous opportunities exist for developing advanced ASO-based therapies of SMA. Specific concerns associated with an ASO-based therapy are the potential off-target effects and the lack of body-wide distribution. Recent reports employing ISS-N1-targeting ASOs support that both of these concerns could be mitigated by optimizing the size, chemistry and terminal modifications of ASOs [43,86]. There are additional avenues to enhance the efficacy of an ASO-based drug of SMA by combining with small molecules that promote *SMN2* transcription and/or *SMN2* exon 7 inclusion [31,161–163]. SMA drug development has come a long way, and the available therapies are literally extending the lifespan of SMA children by converting the severe form of disease into milder ones. This extension of a lifespan offers additional opportunities for advanced treatments with novel drugs that are yet to be developed. Distinct from gene therapy, the advantage of antisense technology is its reliance on the manipulation of an endogenous transcript through a defined target. Hence, the ASO-based approach maintains the natural cap on the levels of transcripts generated from the targeted gene in a tissue-specific manner. Small molecules may offer similar advantage, although target specificity is not guaranteed. Given the diverse options available for the improvement of ASO-based drugs, future of SMA therapy appears promising. To a greater significance, information gleaned from the multifaceted investigations on ISS-N1-targeting ASOs could be utilized for developing ASO-based therapies of a growing number of pathological conditions.

## Data Availability

Data sharing is not applicable to this article as no data were created or analysed in this study.
